# Myco-Biocontrol of Insect Pests: Factors Involved, Mechanism, and Regulation

**DOI:** 10.1155/2012/126819

**Published:** 2012-02-23

**Authors:** Sardul Singh Sandhu, Anil K. Sharma, Vikas Beniwal, Gunjan Goel, Priya Batra, Anil Kumar, Sundeep Jaglan, A. K. Sharma, Sonal Malhotra

**Affiliations:** ^1^Department of Biotechnology, Maharishi Markandeshwar, Mullana, Ambala, Haryana 133203, India; ^2^Department of Bio & Nanotechnology, Guru Jambheshwar University of Science & Technology, Hisar, Haryana 125001, India; ^3^Plant Biotechnology Division, Indian Institute of Integrative Medicine (IIIM), Jammu, J & K 180001, India; ^4^Department of Pulmonary Medicine & Critical Care, Mayo Clinic, Rochester, MN 55905, USA

## Abstract

The growing demand for reducing chemical inputs in agriculture and increased resistance to insecticides have provided great impetus to the development of alternative forms of insect-pest control. Myco-biocontrol offers an attractive alternative to the use of chemical pesticides. Myco-biocontrol agents are naturally occurring organisms which are perceived as less damaging to the environment. Their mode of action appears little complex which makes it highly unlikely that resistance could be developed to a biopesticide. Past research has shown some promise of the use of fungi as a selective pesticide. The current paper updates us about the recent progress in the field of myco-biocontrol of insect pests and their possible mechanism of action to further enhance our understanding about the biological control of insect pests.

## 1. Introduction

Myco-biocontrol is an environmentally sound and effective means of reducing or mitigating insect-pests and its effects through the use of natural enemies. Pest-related damages result in a heavy loss, approximately estimated to be US $10,000 millions annually in agricultural production in the field and storage in India. Myco-biocontrol is the use of fungi in biological processes to lower the insect density with the aim of reducing disease-producing activity and consequently crop damage [1, 2]. All groups of insects may be affected and over 700 species of fungi have been recorded as pathogens. Some of these fungi have restricted host ranges, for example, *Aschersonia aleyrodes *infects only scale insects and whiteflies, while other fungal species have a wide host range, with individual isolates being more specific to target pests. Some species are facultative generalist pathogens, such as *Aspergillus *and *Fusarium*. However, most species are obligate pathogens, often quite specific and rarely found, for example, many species of *Cordyceps.*

Entomopathogens such as *M. anisopliae *and *B. bassiana *are well characterized in respect to pathogenicity to several insects and have been used as myco-biocontrol agents for biological control of agriculture pests worldwide. About 11 companies offer at least 16 products based on the entomopathogenic fungi *B. bassiana *at Columbia. These products are not only used in coffee crop but also in other crops such as bean, cabbage, corn, potato, and tomato. They are used to treat haematophagous insect pests and vectors of diseases like mosquitoes and flies. Under natural conditions, fungi are the frequent and often important natural mortality factor in insect populations. Unlike other potential biocontrol agents, fungi do not have to be ingested to infect their hosts but invade directly through the cuticle, and so can, potentially, be used for control of all insects including sucking insects. 

## 2. Sources of Myco-Biocontrol Agents

A substantial number of mycoinsecticides and mycoacaricides have been developed worldwide since the 1960s. Products based on *Beauveria bassiana* (33.9%) [3], *Metarhizium anisopliae* (33.9%), *Isaria fumosorosea* (5.8%), and *B. brongniartii* (4.1%) are the most common among the 171 products [4]. Approximately 75% of all listed products are currently registered, undergoing registration or commercially available, whereas 15% are no longer available. Insects in the orders Hemiptera, Coleoptera, Lepidoptera, Thysanoptera, and Orthoptera comprise most of the targets. Research has focused on the relatively easily produced asexual spores (conidia) of the hyphomycete genera *Metarhizium *[5], *Beauveria*, *Verticillium*, and *Paecilomyces*. These fungi have a wide host range although there is considerable genetic diversity within species and some clades show a high degree of specificity. For example, *Metarhizium anisopliae *var. *acridum *[6] is only effective against acridid insects (grasshoppers and locusts). The commercial *Beauveria bassiana*-based mycoinsecticides are relatively stable compared with other biological insect control agents for lepidopteran insect pests [7, 8]. 

Major concern about the ill effects of the chemicals pesticides along with advances in biotechnology has promoted search for new and ecofriendly insect control alternatives. Among them biological control is one of the most effective alternative. Biological control can be pronounced to involve the use of biological entities for reducing the damage caused by insect pest population. Very less percentage of known insect species are considered as pests, and the control of these insects has been a major challenge before scientific community since the beginning of the agriculture era. Although effective, chemical pesticides are expensive and provide only temporary relief, as the explosive reproductive and evolutionary capacities of the insect allow them to develop mechanisms resistance to these and other control strategies. Apart from the dangers posed upon human population especially health, they also affect nontarget organism and cause irreversible damage to the environment by disturbing the ecological balance. For biocontrol to become an integral part of the modern agriculture, a few goals must be met such as the selection and development of superior biocontrol agents, the development of fermentation system for biomass production, and the development of formulation and delivery systems, which are compatible with microorganism requirements as well as with common agriculture practices.

Past researches have shown fungi being a potential biological control agent mainly due to their high reproductive capabilities, target specific activity, short generation time, and resting stage or saprobic phase-producing capabilities that can ensure their survival for a longer time when no host is present. Primary requirement for the use of an entomogenous fungus as a myco-biocontrol agent is the susceptibility of the insect on one hand and virulence of the fungus on the other hand. The latter depends on selection of a strain with stable, specific efficacy for a target host. Hence there is an immense potential for genetic improvement of fungi for myco-biocontrol.

Deuteromycetes fungi has a broad host range, and in particular *Metarhizium* and *Beauveria* show particular promise as myco-biocontrol agents and are currently being used as myco-insecticides.* Beauveria bassiana *and *Metarhizium anisopliae *are among the first entomopathogenic fungi being successfully used for the myco-biocontrol of insect pests. Molecular techniques for genetic engineering for filamentous fungi provide new opportunities for the study of fungi used in myco-biocontrol of insect pests. The isolation of gene encoding pathogenesis and virulence allows rigorous testing of their role in pathogenesis and should provide a rational basis for strain improvement.

The studies on the fungal pathogenesis for myco-biocontrol of insect pests are still at the preliminary stages. However, the development of molecular biological technique for entomopathogenic fungi such as *Beauveria bassiana *and *Metarhizium anisopliae, *which are coupled with cloning of genes encoding putative pathogenesis determinants, will create more potential candidates to manage the notorious insect pests population. Research in this field will inevitably depend on the development if sensitive techniques for monitoring the environmental fate of recombinant strain for management of insect pests are designed. 

## 3. Entomopathogenic Fungi

Entomopathogenic fungi are among the first organisms to be used for the biological control of pests. More than 700 species of these fungi from around 90 genera are pathogenic to insects. Most of them are found within the deuteromycetes and entomophthorales. Some entomopathogenic fungi have restricted host ranges, for example, *Aschersonia aleyrodes *infects only scale insects, and whiteflies, while other fungal species have a wide host range, with individual isolates being more specific to target pests. Entomopathogens such as *M. anisopliae *and *B. bassiana *are well characterized in respect to pathogenicity to several insects, and they have been used as agents for the biological control of agriculture pests worldwide. About 11 companies offer at least 16 products based on the entomopathogenic fungi *B. bassiana*. These products are not only used in the coffee crop but also in other crops such as bean, cabbage, corn, potato, and tomato. They are also used to treat haematophagous insect pests and vectors of diseases like mosquitoes and flies [9]. Biopesticide has very complex mode of action unlike chemical pesticides, therefore resistance in pest could not be developed. 

## 4. Bio-Management  of Insect-Pests  by Entomopathogenic  Fungi

The entomogenous word has been derived from two Greek words, “*entomon*” meaning insects and “*genes*” meaning arising in. Therefore, the etymological meaning of entomogenous microorganism is “microorganisms which arise in insects.” The power of these entomogenous microorganisms in bringing about a certain degree of natural or microbial control of insect pests is directly related to human welfare which has attracted the attention of microbiologist, molecular biologists, and entomologists in the recent years. Several entomopathogens, when inundatively introduced into a variety of habitats, can provide effective long-term to short-term control.  Table 1 gives an overview of different types of organisms, including pathogenic microorganisms such as viruses, bacteria, fungi, protozoa, and nematodes for their use as biocontrol agents. The most propitious integration of pathogens, predators, insect's growth regulators, and conventional insecticides may provide us with long-term control of serious agricultural insect pests. Some of the potential candidates for myco-biocontrol of insect pests are discussed here.

### 4.1. *Beauveria* sp

*Beauveria bassiana*, a filamentous fungus, belongs to a class of insect pathogenic deuteromycete also known as imperfect fungus. Strains of *Beauveria *are highly adapted to particular host insects. A broad range of *B. bassiana *spp. have been isolated from a variety of insect worldwide which are of medicinal or agricultural importance. *Beauveria bassiana* is a fungus that grows naturally in soils throughout the world and acts as a pathogen on various insect species, causing white muscardine disease, therefore belongs to the entomopathogenic fungi [10–14]. An interesting feature of *Beauveria *sp. is the high host specificity of many isolates. Hosts of medicinal importance include vectors for agents of tropical infectious diseases such as tsetse fly *Glossina morsitans*, and sand fly *Phlebotomus *that transmits *Leishmania *and bugs of genera *Triatoma *and *Rhodnius*, the vectors of Chagas disease. Hosts of agricultural and forest significance include the Colorado potato beetle, the codling moth, and several genera of termites, American bollworm *Helicoverpa armigera *[15], *Hyblaeapara* and *Eutectona machaeralis*. Furthermore, the high level of persistence in the host population and in the environment provides long-term effects of the entomopathogenic fungi on pest suppression, if an epizootic is caused. It is being used as a biological insecticide to control a number of pests such as termites, whitefly, and in malaria-transmitting mosquitoes [16, 17]. *B. bassiana* is the anamorph (asexually reproducing form) of *Cordyceps bassiana*. The latter teleomorph (the sexually reproducing form) has been collected only in eastern Asia [3]. Rehner and Buckley [18] have shown that *B. bassiana* consists of many distinct lineages that should be recognized as distinct phylogenetic species. This ubiquitous fungus has long been known to be the most common causative agent of disease associated with dead and moribund insects in nature [19] and has been scrutinized worldwide as a microbial control agent of hypogeous species [20]. Many curculionidae weevils with a sub-terranean larval stage are highly susceptible to this white muscardine disease [21]. Like many species of entomogenous fungi, *B. bassiana* is composed of many genetically distinct variants associated with geographical location and host which differ substantially in their ability to produce pathogenesis. As an insecticide, the spores are sprayed on affected crops as an emulsified suspension or wettable powder. *B. bassiana* parasitizes a very wide range of arthropod hosts and therefore is considered as a nonselective biological insecticide. *B. bassiana *is also applied against the European corn borer *Ostrinia Mubilalis*, pine caterpillars *Dendrolimus *spp., and green leafhoppers *Nephotettix *spp.

### 4.2. *Verticillium lecanii*

Another entomopathogenic fungus *Verticillium lecanii* is a widely distributed fungus, which can cause large epizootic in tropical and subtropical regions, as well as in warm and humid environments [22]. It was reported by Kim et al. [23] that *V. lecanii *was an effective biological control agent against *Trialeurodes vaporariorum* in South Korean greenhouses. This fungus attacks nymphs and adults and stucks to the leaf underside by means of a filamentous mycelium [22]. In 1970s, *Verticillium lecanii *was developed to control whitefly and several aphids species, including the green peach aphids (*Myzus persicae*) for use in the greenhouse chrysanthemums [16]. 

*Verticillium lecanii* was considered as a major parasite which caused a massive decline of cereal-cyst nematode populations in monocultures of susceptible crops [24]. *Verticillium chlamydosporium *has a wide host range amongst cyst and root-knot nematodes but it is very variable and only some isolates may have potential as commercial biological control agents.

### 4.3. *Metarhizium* spp

*Metarhizium anisopliae* is also very potential pathogen on insect pests and is explored for myco-biocontrol of notorious insect pests [10, 25]. A complete bioactivity of* M. anisopliae* has been tested on teak skeletonizer *Eutectona machaeralis* and found *M. anisopliae *to be a potential myco-biocontrol agent of teak pest [26]. Hasan et al. [27] have tested spore production of* M. anisopliae* by solid state fermentation.

### 4.4. *Nomuraea* sp

*Nomuraea rileyi *another potential entomopathogenic fungi is a dimorphic hyphomycete that can cause epizootic death in various insects. It has been shown that many insect species belonging to Lepidoptera including *Spodoptera litura *and some belonging to *Coleoptera *are susceptible to *N. rileyi *[28]. The host specificity of *N. rileyi *and its ecofriendly nature encourage its use in insect pest management. Although, its mode of infection and development have been reported for several insect hosts such as *Trichoplusia ni*, *Heliothis zea*, *Plathypena scabra*, *Bombyx mori*, *Pseudoplusia includes,* and *Anticarsia gemmatalis*. Another insect *Spilosoma* was found to be severely attacked by *Nomuraea rileyi*, hence studied in detail for its myco-biocontrol [29]. Similarly an epizootic of *Nomuraea rileyi* was observed on *Junonia orithya* [30] which was proved to be the best alternative to manage the hedge plant eater *Junonia orithya*.

### 4.5. *Paecilomyces* sp

*Paecilomyces* is a genus of nematophagous fungus which kills harmful nematodes by pathogenesis, causing disease in the nematodes. Thus, the fungus can be used as a bionematicide to control nematodes by applying to soil (Table 2). *Paecilomyces lilacinus* principally infects and assimilates eggs of root-knot and cyst nematodes. The fungus has been the subject of considerable biological control research following its discovery as a biological control agent in 1979. *Paecilomyces fumosoroseus* (Wize) Brown and Smith [31] (Hyphomycetes) is one of the most important natural enemies of whiteflies worldwide, and causes the sickness called “Yellow Muscardine” [22]. Strong epizootic potential against *Bemisia *and *Trialeurodes *spp. in both greenhouse and open field environments has been reported. *P. lilacinus* has been considered to have the greatest potential for application as a biocontrol agent in subtropical and tropical agricultural soils. The ability of this fungus to grow extensively over the leaf surface under humid conditions is a characteristic that certainly enhances its ability to spread rapidly through whitefly populations [32]. 

Natural epizootics of these fungi suppress *Bemisia tabaci* populations. Epizootics caused by *Paecilomyces fumosoroseus* also lead to substantially reductions in *B. tabaci* populations during or immediately following rainy seasons or even prolonged periods of cool, humid conditions in the field or greenhouse [4]. However, in general, epizootics of naturally occurring fungi cannot be relied upon for control. Only a few species of fungi have the capacity to cause high level of mortality, and development of natural epizootics which is not only dependent on the environmental conditions, but also influenced by various crop production practices. Also, epizootics often occur after intense injury has already been inflicted by whiteflies [4]. Kim et al. [23] reported that *P. fumosoroseus *is best for controlling the nymphs of whitefly. These fungi cover the whitefly's body with mycelial threads and stick them to the underside of the leaves. The nymphs show a “feathery” aspect and are surrounded by mycelia and conidia [22]. *P. furiosus* is also used to control mosquito sp. *Culex pipiens* [25].

## 5. Mode of Action of Entomopathogenic Fungus

Entomopathogenic fungi constitute the largest single group of insect pathogens among microorganisms. Such insect killing fungi are very fast Microorganisms to be recognized as disease causing agent in insects. Entomogenous fungi are promising myco-biocontrolling agent for a number of crop pests. Several species belonging to order Lepidoptera, Coleoptera, Homoptera, Hymenoptera, and Diptera are susceptible to various fungal infections. Entomopathogenic fungi have a great potential as myco-biocontrol agents, as they constitute a group with over 750 species that, when dispersed in the environment, provoke fungal infections in insect populations. 

## 6. The Infection Process

Fungi have an unique mode of infection; they reach the haemocoel through the cuticle or possibly through the mouth parts. Ingested fungal spores do not germinate in the gut and are voided in the faeces. The death of the insect results from a combination of factors: mechanical damage resulting from tissue invasion, depletion of nutrient resources and toxicosis, and production of toxin in the body of insect.

**Conidial Attachment with the Cuticle**

For most of the entomopathogenic fungi host location is a random event and attachment is a passive process with the aid of wind or water. Attachment of a fungal spore to the cuticle surface of a susceptible host represents the initial event in the establishment of mycosis. It was observed that dry spores of *B. bassiana *possess an outer layer composed of interwoven fascicles of hydrophobic rodlets. This rodlet layer appears to be special to the conidial stage and has not been reported on the vegetative cells. The adhesion of dry spores to the cuticle was suggested to be due to nonspecific hydrophobic forces imposed by the rodlets [33]. Some of these moieties like lectins, a kind of carbohydrate binding glycoproteins, have also been detected on the conidial surface of *B. bassiana*. It was also observed that lectins could be involved in binding between conidia and the insect cuticle. The exact mechanisms responsible for the interaction between fungal spores and the cuticle remain to be determined [34]. When the pathogen reaches and adheres to the host surface, it proceeds with rapid germination and growth which are profoundly influenced by the availability of water, nutrients, oxygen as well as pH, and temperature, and by the effects of toxic host-surface compound. Fungi with a broad host range germinate in culture in response to a wide range of nonspecific carbon and nitrogen sources [35]. Entomopathogenic fungi with restricted host range appear to have more specific requirements for germination [36].

**Formation of an Infection Structure**

**Figure 1:**
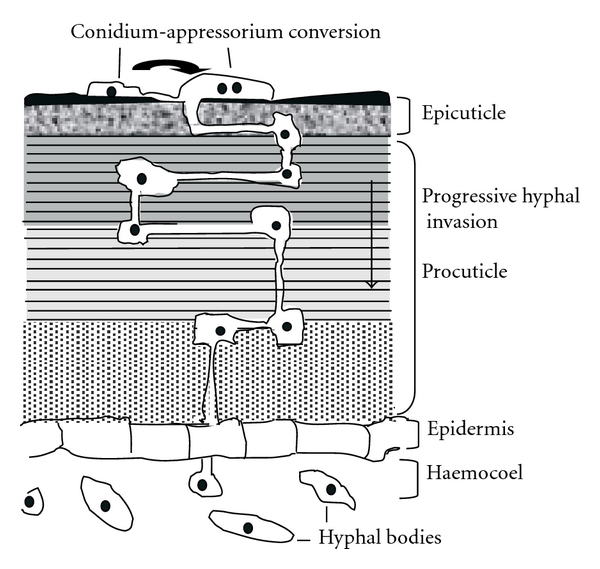
Depiction of the infection process in B. bassiana: structure of the insect cuticle and mode of penetration of fungal hyphae. Formation of the appressorium from the conidia helps in cuticle invasion and subsequent hyphal penetration to the haemocoel. Tissue invasion by hyphae and proliferation of hyphal bodies leads to insect death.

Entomopathogenic fungi invade their hosts by infection process: penetration of the host cuticle or put pressure on cuticle by making appressorium and then penetrate by penetration peg [35]. The cuticle has two layers: the outer epicuticle and the procuticle. The epicuticle is a very complex thin structure that lacks chitin but contains phenol-stabilized proteins and is covered by a waxy layer containing fatty acids, lipids and sterols [37]. The procuticle forms the majority of the cuticle and contains chitin fibrils embedded into a protein matrix together with lipids and quinones [38]. Protein may account for up to 70% of the cuticle. In many areas of the cuticle, the chitin is organized helically giving rise to a laminate structure. Entomopathogenic fungi, *B. bassiana *conidia germinate on the host surface and differentiate an infection structure termed appressorium. The appressorium represents an adaptation for concentrating physical and chemical energy over a very small area so that access may be achieved efficiently (Figure 1). Thus, formation of the appressorium plays a pivotal role in establishing a pathogenic interaction with the host. Appressorium formation may be influenced by host surface topography, and biochemical investigations indicate the involvement of the intracellular second messengers Ca^2+^ and cyclic AMP (cAMP) in appressorium formation [39] or in general when the cuticle in hard [35].

**Penetration of the Cuticle**

Entomopathogenic fungi need to penetrate through the cuticle into the insect body to obtain nutrients for their growth and reproduction. Entry into the host involves both enzymic degradation and mechanical pressure as evidenced by the physical separation of lamellae by penetrated hyphae. A range of extracellular enzymes that can degrade the major components of insect cuticle, including chitinases, lipases, esterases and at least four different classes of proteases, have been suggested to function during the fungal pathogenesis. Although the complex structure of the insect cuticle suggests that penetration would require the synergistic action of several different enzymes, much of the attention has focused on the cuticle-active endoprotease as a key factor in the process.

The production of cuticle-degrading enzymes by *M. anisopliae *during infection structure formation on *Calliphora vomitoria *and *Manduca sexta *has been investigated by biochemical and histochemical analyses both *in vivo *and *in vitro*. Among the first enzymes produced on the cuticle are endoproteases (termed PR1 and PR2) and aminopeptidases, coincident with the formation of appressoria. N-Acetylglucosaminidase is produced at a slow rate as compared to the proteolytic enzymes [40].

These fungi begin their infective process when spores are retained on the integument surface, where the formation of the germinative tube initiates, the fungi starts excreting enzymes such as proteases, chitinases, quitobiases, lipases, and lipoxygenases. *V. lecanii *is capable of penetrating the insect cuticle only with its germ tube while *M. anisopliae *and *B. bassiana *produce specific infection hyphae originating at appressoria. After the successful penetration, the fungus is then distributed into the haemolymph by formation of blastospores [41].

Different works are going on all over the world to distinguish the various enzymes which are required for the mechanism of entomopathogenic *Metarhizium anisopliae, M. flaviviridae*,* Paecilomyces farinosus, Beauveria bassiana*, and *B. brongniartii*. Host specificity may be associated with the physiological state of the host system (i.e., insect maturation and host plant) [42], the properties of the insect's integument with the nutritional requirements of the fungus [43], and the cellular defense of the host [44]. In contrast to bacteria and viruses that pass through the gut wall from contaminated food, fungi have a unique mode of infection. They reach the haemocoel through the cuticle. 

## 7. Production of Toxins

A plethora of work with circumstantial evidence is available from deuteromycete pathogens for the involvement of fungal toxins in host death. The action of cytotoxins is suggested by cellular disruption prior to hyphae penetration. Behavioural symptoms such as partial or general paralysis, sluggishness, and decreased irritability in mycosed insects are consistent with the action of neuromuscular toxins [45]. *B. bassiana *and *M. anisopliae *produced significant amounts of toxic compounds within their hosts. For example, the toxins Beauvericin, Bassianolide, Isarolides, and Beauverolides have been isolated from *B. bassiana *infected hosts [46, 47], toxins Destruxins (DTXs) and Cytochalasins have been isolated from *M. anisopliae* infected hosts. The toxins have shown to have diverse effects on various insect tissues. DTX depolarizes the *lepidopteran *muscle membrane by activating calcium channels. In addition, function of insect hemocytes can be inhibited by DTX [48]. Presumably, there are still many toxins that remain to be isolated from parasitized insects and except DTXs, their relevance in the process of pathogenicity remains to be studied in detail.

## 8. Genetic  Engineering Studies  of  Entomopathogenic Fungi

A more widespread use of fungi for myco-biocontrol will depend on improvements of wild-type strains by combining characteristics of different strains and mutants. Two types of improvements could be considered: (i) improving the efficacy of the insecticide, by reducing the dose necessary to kill the insects, by reducing the time to kill the pest or decreasing crop damage caused by the pest by reducing the feeding time; (ii) expanding the host range. Essential for the development of a hypervirulent strain is a complete understanding of the remarkable pathology of fungal infections. Molecular biology provides the necessary tools for dissecting the mechanisms of pathogenesis and in the longer term for producing recombinant organisms with new and relevant characteristics. Initial development towards these goals has occurred with *M. anisopliae *and to a much lesser extent with *B. bassiana *[49]. Genetic transformation systems, which are an essential part of modern fungal research, and are necessary for the experimental manipulation of virulence genes *in vitro *and *in vivo, *have been established [27, 50, 51]. The success of utilizing these procedures depends on the availability of selectable transformation markers [26]. Transformation techniques have been used to isolate specific pathogenic genes, investigate virulence determinants of *M. anisopliae *and *B. bassiana*, and to produce a strain with enhanced virulence. Unravelling the molecular mechanisms of fungal pathogenesis in insects will provide the basis for the genetic engineering of entomopathogenic fungi.

## 9. Molecular  Studies  of  Entomopathogenic Fungi

Implementation of PCR-based tools for characterization of organisms has greatly advanced the understanding of the phylogenies and species in entomopathogenic fungi, especially in *B. bassiana *and *M. anisopliae*. These fungi have received a lot of interest due to their potential as biocontrol agents of pests. A number of unspecific DNA-based methods have been used specially in *Beauveria *[52]. Random amplified polymorphic DNA (RAPD) has been used in many studies. It is based on the use of short general primers that anneal to unspecified regions in the template DNA whereas universally primed (UP) PCR is based on longer general primers and a higher annealing temperature which makes it more robust in terms of reproducibility [53–56]. UP-PCR has been used to separate sympatric isolates of *Beauveria *in Denmark and was used to place isolates in genetic groups [57]. For ecological studies, random amplified polymorphic DNA (RAPD) was used in combination with specific methods to separate isolate genotypes of *M. anisopliae *from Canadian soil [58] and to relate these genotypes to the origin of isolation. Thakur et al. [13] studied forty-eight isolates of indigenous strains of *B. bassiana *collected from Central India employing protease zymography and RAPD analysis. High genetic and biochemical diversities were indicated with a clear group of strains from Lepidopteran and Coleopteran insect hosts. 

Different strategies have also been used for the analysis-RFLPs (restriction Fragment Length Polymorphism), AFLPs (amplified fragment length polymorphism). Digestion of PCR products of specific DNA regions, such as genes or ITS (internal transcribed spacer), with restriction enzymes, yields fragments of variable sizes. These RFLPs have been used for the characterization of both *Beauveria *and *Metarhizium *species [58]. *B. bassiana *and *M. anisopliae *[59–61] have been characterized using AFLP (amplified fragment length polymorphism), inter-simple-sequence repeats (ISSR), simple sequence repeats (SSRs), or microsatellites. Internal transcribed spacer sequences (ITSs) have been widely used in fungal systematics [6, 62]. In the case of the genus *Paecilomyces*, the analysis of sequences of the large and the small subunit rRNA gene has already indicated the polyphyly of the genus [63, 64]. Recent development of microsatellite markers [65, 66] has surely provided an insight in the population ecology of *B. bassiana* and *M. anisopliae*. *B. bassiana* has been linked to plants as an endophytic fungus [67], and *M. anisopliae *has been shown to be associated with the rhizosphere of plants [68]. EST (expressed sequence tag) analysis of entomopathogenic fungus *Beauveria* (*Cordyceps*) *bassiana *has been studied using cDNA libraries [69]. EST analyses of two subspecies of *M. anisopliae* revealed distinct patterns of expression of proteases and pathogenicity factors. These expression patterns have led to the ability to examine gene expression during infection of various insect hosts [70, 71].

## 10. Myco-Biocontrol Agents

The advantages of using fungi as myco-biocontrol agents are as follows (1) Their high degree of specificity for pest control. Fungi can be used to control harmful insect pests without affecting beneficial insect predators and nonharmful parasites. (2) The absence of effects on mammals and thus the reduction of the hazards normally encountered with insecticide applications, such as pollution of the environment. (3) The lack of problems caused to insect resistance and prolonged pest control. (4) A high potential for further development by biotechnological research. (5) High persistence in the environment provides long-term effects of entomopathogenic fungi on pest suppression. However, there are also a number of constraints on the use of fungi as insecticides: (1) 2-3 weeks are required to kill the insects whereas chemical insecticides may need only 2-3 hours. (2) Application needs to coincide with high relative humidity, low pest numbers, and a fungicide free period (3) Due to the high specificity additional control agents are needed for other pests. (4) Their production is relatively expensive and the short shelf life of spores necessitates cold storage. (5) The persistence and efficacy of entomopathogenic fungi in the host population varies among different insects species, thus insect-specific application techniques need to be optimised to retain long-term impacts. (6) A potential risk to immunosuppressive people.

Entomopathogenic fungi are important as they are virulent, infect by contact, and persist in environment for a long period of time. These can be mass produced in liquid or solid media. Most of the entomopathogenic fungi are facultative parasites which exist as saprotrophs and therefore can be grown apart from living hosts. Few groups are obligate parasites which must be reared in living hosts. Introduction of fungal pathogens into the host population initiates epizootic and prevents or reduces damage by the pest. The initiation of artificial epizootics has been accomplished for long-term control especially in areas where high humidity condition prevails. There are several defense mechanisms in insect which prevents the penetration and the growth of the fungus. The most common is the melanisation of the cuticle at infection site. Entomopathogenic fungi can display either a very broad host spectrum like *M. anisopliae, B. bassiana *or have a very narrow host range like *Aschersonia *spp. [41].

## 11. Conclusions

Modern techniques in genetic engineering and biotechnology are extremely helpful in manipulating the desired traits in entomopathogenic fungi which can further improve its bioactivity. Numerous advantages one can foresee of using these fungal pathogens as pest control agents. They are host-specific having a wide host range and more importantly being less toxic to animals. Biological control agents have shown a lot of promise in terms of activity, though its efficacy is affected by many factors such as biotic and nonbiotic factors, host plant, and at the level of nematode infestation. There is a strong urge to elucidate the essence of these factors to improve the overall efficacy of these control agents along with developing novel methods to deliver sufficient inoculum at the target sites. Modern techniques in biotechnology has the potential to manipulate desirable traits of these entomopathogenic fungi to improve the overall field activity.

## Figures and Tables

**Table 1:** Different types of organisms including pathogenic microorganisms such as viruses, bacteria, fungi, protozoa, and nematodes have been used as biocontrol agents.

Biocontrol agent	Common examples	Biological action
*Parasitic insects*: living organisms remaining in close association with their hosts and gradually derive their food from the host	*Trichogramma chilonis, Epiricania melanoleuca *	They live and feed internally or externally on the host.
* Predatory insects*		
	*Chrysoperla carnea*, *Cryptolaemus montrouzieri *	Insects which kill and devour the prey
*Microorganisms *(Bacteria, fungi, viruses)		
	Bacteria, for example, *Bacillus thuringiensis *Fungi, for example, species of *Trichoderma, Nomuraea, Paecilomyces,Verticillium, Metarhizium, and Beauveria *Viruses, for example, nuclear polyhedrosis virus	Cause diseases in pests and inhibit the harmful fungi

**Table 2:** Various bioactive products derived from entomopathogenic fungi which could be commercially useful for field application have been illustrated.

Product	Fungus	Biological action
Mycotal	*Verticillium lecanii*	Fungal pesticide
Pfr21	*Paecilomyces fumosoroseus *	Fungal pesticide
Verelac	*Verticillium lecanii*	Sucking pests
Beevicide	*Beauveria bassiana*	Borer type pests
Grubkill	Selected fungus and bacteria	Borers and sucking pest
Pelicide	*Paecilomyces lilacinus*	Effective against nematode
Biologic Bio 1020	*Metarhizium anisopliae*	Mycelium granules as pesticide
Bioter	*Verticillium lecanii*	Effective against termites
Brocaril	*Beauveria bassiana*	Wettable powder used as pesticide
Ostrinil	*Beauveria bassiana*	Microgranules of mycelium used as pesticide
Boverol	*Beauveria bassiana*	Dry pellets as pesticide
		
Naturalis	*Beaveria bassiana*	Liquid formulation as pesticide
Mycontrol-WP	*Beauveria bassiana*	Wettable powder as pesticide
Betel	*Beauveria brongniartii*	Microgranules of mycelium used as pesticide
Engerlingspilz	*Beauveria brongniartii*	Barley kernels colonized with fungus used as pesticide
Biopath	*Metarhizium anisopliae*	Conidia on a medium used as pesticides
Biomite	*Verticillium lecanii* and other entomopathogenic organisms	Effective against mites
Biogreen	*Metarhizium anisopliae*	Conidia produced on grain used as pesticide
Naturalis-O and BotaniGard	*Beauveria bassiana*	Effective against whiteflies
Trypae Mix	*Trichoderma* and *Paecilomyces *	Effective against fungal pathogens and nematodes in soil
